# Novel Therapeutic Delivery of Nanocurcumin in Central Nervous System Related Disorders

**DOI:** 10.3390/nano11010002

**Published:** 2020-12-22

**Authors:** Elisa Panzarini, Stefania Mariano, Stefano Tacconi, Elisabetta Carata, Ada Maria Tata, Luciana Dini

**Affiliations:** 1 Departament of Biological and Environmental Sciences and Technologies (Di.S.Te.B.A.), University of Salento, 73100 Lecce, Italy; elisa.panzarini@unisalento.it (E.P.); stefania.mariano@unisalento.it (S.M.); stefano.tacconi@unisalento.it (S.T.); elisabetta.carata@unisalento.it (E.C.); 2 Departament of Biology and Biotechnology “C. Darwin”, Sapienza University of Rome, 00185 Rome, Italy; adamaria.tata@uniroma1.it; 3 CNR Nanotec, Campus Ecotekne, University of Salento, 73100 Lecce, Italy

**Keywords:** nanomaterials, curcumin, drug delivery, blood brain barrier, glioblastoma, neurological disorders

## Abstract

Nutraceuticals represent complementary or alternative beneficial products to the expensive and high-tech therapeutic tools in modern medicine. Nowadays, their medical or health benefits in preventing or treating different types of diseases is widely accepted, due to fewer side effects than synthetic drugs, improved bioavailability and long half-life. Among herbal and natural compounds, curcumin is a very attractive herbal supplement considering its multipurpose properties. The potential effects of curcumin on glia cells and its therapeutic and protective properties in central nervous system (CNS)-related disorders is relevant. However, curcumin is unstable and easily degraded or metabolized into other forms posing limits to its clinical development. This is particularly important in brain pathologies determined blood brain barrier (BBB) obstacle. To enhance the stability and bioavailability of curcumin, many studies focused on the design and development of curcumin nanodelivery systems (nanoparticles, micelles, dendrimers, and diverse nanocarriers). These nanoconstructs can increase curcumin stability, solubility, in vivo uptake, bioactivity and safety. Recently, several studies have reported on a curcumin exosome-based delivery system, showing great therapeutical potential. The present work aims to review the current available data in improving bioactivity of curcumin in treatment or prevention of neurological disorders.

## 1. Introduction

Natural products have been used for a long time and are still used by people for health improvement and for the treatment of various diseases. In 1989, Dr Stephan De Felice, President of the Foundation for Innovation in Medicine (FIM), coined the word nutraceuticals by the combination of “nutrition” and “pharmaceutical” and defined its concept as “*a food or part of a food that provides medical or health benefits, including disease prevention and/or treatment*” [1]. Thus, nutraceuticals are all the substances extracted from natural sources, derivatives from human metabolism (dehydroepiandrosterone or DHEA, S-adenosylmethionine or SAMe, carnitine, creatine, coenzyme Q10, lipoic acid, melatonin) and bioactive plant dietary components (polyphenols, saponins, probiotics, phytoestrogens, dietary fibers, carotenoids) that preserve their original properties without any chemical manipulation. Nutraceuticals are marketed as dietary supplements (e.g., vitamins, minerals, co-enzyme Q, carnitine), functional foods (e.g., yogurts, cereals, snacks, etc.) and medicinal foods (e.g., health bars with added medications, transgenic cows and lacto-ferrin for immune enhancement, etc.) that have health or medical benefits in disease prevention and treatment [2]. Health benefits of nutraceuticals rely on good bioavailability, long half-life and absence of side effects with respect to synthetic drugs, beside the enhancement of physiological processes, for example, immune response [3].

Among the large amount of nutraceuticals, considerable attention is given to turmeric derivatives due to a plethora of biological benefits (i.e., antioxidant, anti-inflammatory, anti-cancer, anti-growth, anti-arthritic, anti-atherosclerotic, anti-depressant, anti-aging, anti-diabetic, anti-microbial, wound healing, memory-enhancing, chemopreventive, chemosensitization and radiosensitization) [4,5,6]. Curcumin is the most widely studied turmeric derivative and represents 60–70% of the curcuminoids extracted from the rhizome of *Curcuma longa* [7]. Curcumin, with its characteristic yellow color, is a polyphenol compound with numerous bioactive properties for which it has been used for multiple applications in traditional medicine for more than 2000 years [8]. Given the many biological targets of curcumin, its effects in different biological pathways and processes have been well characterized, including adhesion molecules, transcription factors, growth factors, inflammatory mediators, apoptotic regulators, enzymes, kinases, membrane receptors and antioxidant systems [9]. Together with the fact that curcumin has little to no side effects, neuroprotective properties are also reported. Therefore, there is a particular growing interest about curcumin’s impact in central nervous system (CNS)-related neurodegenerative disorders, such as Alzheimer’s and Parkinson’s diseases, and brain malignancies, such as glioblastoma multiforme (GBM) [10,11].

Despite these amazing properties, the utility of curcumin is greatly hindered by its weak absorption, rapid metabolism, systemic elimination, degree of water solubility and limited blood brain barrier (BBB) permeability [12]. Therefore, various strategies have been proposed to overcome these limitations, like the use of adjuvants, nanomaterials, curcumin phospholipid complexes and curcumin formulated with various oils or metabolism inhibitors [13,14,15,16].

One of the emerging strategies in terms of improvement of the bioavailability and efficacy of curcumin is the development of nano-delivery. Indeed, many curcumin nano-delivery systems are currently under investigation. Recently, exosomes, nano-sized vesicles physiologically produced by cells, have been successfully proposed as curcumin delivering systems for the treatment of CNS-related pathologies, for their excellent biodegradability, biocompatibility and ability to cross the BBB [17].

The consumption of nutraceuticals is growing strongly in Europe, USA and Asia, mainly in the form of functional beverages, to contribute to the welfare of the increasing elderly population and, thus, to counter the increase in metabolic diseases. The most competitive companies involved world wide in the nutraceutical markets are exploring new field of application and new technological process of preparation to improve activities of compounds. Here, the current state of the art in nano-sized constructs able to improve bioactivity of curcumin in nervous system disorders is reviewed.

## 2. Bioactivity and Health Benefits of Curcumin

Curcumin was firstly described by Vogel and Pelletier in 1815, as a mix of resin and turmeric oil, while the pure form was obtained in 1842 by Vogel Jr. The chemical structure of curcumin was defined 68 years later by Milobedzka and Lampe in (1E, 6E)-1,7-bis (4-hydroxy-3 methoxyphenyl) [14,18]. Curcumin belongs to the polyphenols family, and it represents the main curcuminoid of turmeric *Curcuma longa* extract [19]. The typical composition of commercial curcumin is a combination of curcumin (~77%), demethoxy curcumin (~17%) and bisdemethoxy curcumin (~3%). Curcumin finds large use as food ingredient, as cosmetic industrial dye and in the formulation of medicinal products (e.g., to relieve muscle pain, inflammation, rheumatoid arthritis, inflammatory and gastrointestinal disorders, intermittent fever and leukoderma).

Important biotechnological applications of curcumin are widely used in the food industry where it is used as a spice or food additive, at a dose of 5–500 mg/kg, to ameliorate foodstuffs’ palatability and storage stability. The European Union has authorized the use of curcumin as food color with the name of CI 75300, Natural Yellow 3 or diferuloylmethane, and with the E100 code [20]. Curcumin is an efficient food preservative in its ability to suppress lipid peroxidation, suggesting a role as a possible natural preservative [21]. At a concentration of 1–2%, curcumin showed antimicrobial activity preserving stored chicken meal from contamination for about 90 days [22]. In the cosmetics sector curcumin is widely used for its antioxidant and anti-inflammatory activity and skin lightening property. In fact, in in vitro curcumin treated cells, including epidermal and dermal layers, inhibition of collagenase, hyaluronidase and elastase was demonstrated [23]. Altered skin pigmentation (such as solar elastoses, solar lentigines, actinic keratosis) caused by photodamage is reduced after curcumin treatment [23]. Curcumin essential oil has dermo-protective properties and stabilizes fatty components of cosmetics; it is, thus, used in the formulations of soaps and cosmetics, and to prevent the rancidity of lipids [24]. The yellow color of curcumin makes it an ideal natural dye in hair coloring moisturizers. The numerous recognized health benefits of curcumin, namely, anti-diabetic, anti-tumor and hepato-, neuro- and cardiovascular protective effects, depend on its metabolism that affects antioxidant, anti-inflammatory and immunoregulatory activities [25]. Curcumin can protect against inflammation by decreasing levels of pro-inflammatory cytokines, such as tumor necrosis alpha (TNFα), interleukin-6 (IL6) and interleukin 1β (IL1β), increasing levels of the anti-inflammatory cytokines interleukin-10 (IL10) and transforming growth factor- beta (TGFβ) [26] and by acting on NF-kB and peroxisome proliferator-activated receptor-gamma (PPAR-γ) pathways and myeloid differentiation protein 2-TLR4 co-receptor (TLR4-MD2) signaling [27,28,29].

**Figure 1:**
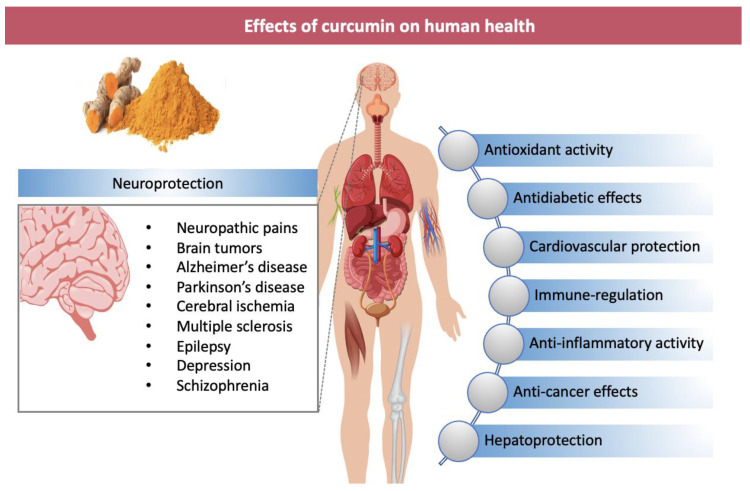
Biological activities of curcumin and related benefits on health. Curcumin finds application in management of central nervous system pathologies (neurodegenerative disorders, brain cancer and health mental illness).

Finally, numerous preclinical studies have suggested neuroprotection properties of curcumin in treating Alzheimer’s disease (AD), Parkinson’s disease (PD), amyotrophic lateral sclerosis (ALS), multiple sclerosis (MS), epilepsy, stroke, traumatic brain injury, spinal cord injury, depression, dementia, schizophrenia and brain tumors [10,11,12] (Figure 1).

The imbalance between reactive oxygen species (ROS) production and antioxidant enzymes activity causes oxidative stress, which is associated with various chronic diseases. The presence of an electron-donating phenolic hydroxyl group in the chemical structure of curcumin confers antioxidant activity [30]. The scavenger activity of curcumin reduces oxidative stress and prevents ROS formation, namely, superoxide and nitric oxide radicals and hydrogen peroxide [31]. Curcumin prevents oxidative damage by blocking nuclear factor kB (NF-kB) activation. In human L02 hepatocytes pretreated with curcumin ROS formation caused by quinocetone was inhibited [32]. Curcumin administration restores the DNA-methyltransferase function in diabetic mice and inhibits hyperglycemia-induced ROS production and, in obese patients, reduces oxidative stress [33,34,35]. The activity of several antioxidant enzymes, (paraoxonase 1 arylesterase, catalase, CAT; glutathione S-transferase, GST; glutathione peroxidase, GSH-Px; superoxide dismutase, SOD; heme oxygenase-1, OH-1) is enhanced by curcumin that, by reducing lipid peroxidation, protect against carcinogenesis processes [36,37,38]. It should be kept in mind that the antioxidant effect of curcumin depends on the dose used and on the presence in culture medium of metal ions and that an antioxidant in vitro effect could not have the same in vivo effect [39,40,41].

Curcumin is also beneficial for the immune system by interacting with immune cells (T and B lymphocytes, macrophages, dendritic cells and natural killer cells) by modulating IgG, IgM, IgA and immune mediators (IL-1β, IL-4, IL-6, IL-17A, TNF-α) and thus, protecting against immune-related diseases [42]. It has been demonstrated that curcumin reduces the number of neutrophils and eosinophils and increases the amounts of lymphocytes. For example, curcumin regulates Th1/Th2 balance in ovalbumin-sensitized rats by stimulating Th1 cells and inhibiting Th2 cells [43]. Curcumin stimulates differentiation of bone marrow-derived mesenchymal stem cells towards anti-inflammatory M2 macrophages, providing a favorable microenvironment for adult full-thickness cutaneous wound healing [44]. Moreover, curcumin decreases macrophage infiltration and inhibits NF-kB pathway in macrophages [45].

Several chronic diseases, such as autoimmune, cardiovascular, endocrine, neurodegenerative and cancer, are characterized by a chronic inflammatory outbreak closely related to oxidative stress [25]. The effectiveness of curcumin in the prevention and treatment of these diseases, as well as metabolic, neurological, skin and infectious diseases, has been demonstrated in several preclinical and clinical studies [46].

## 3. Curcumin and CNS-Related Pathologies

### 3.1. Neurodegenerative Pathologies

In the last decades, a lot of attention was paid to increasing chronic neurodegenerative disorders. The population affected grows progressively and the impact of these diseases on patients’ lives, on society and the economy is more relevant [47]. Growing evidence indicates that oxidative stress contributes to the etiology and the progression of nervous system disorders, such as AD, PD, ALS and MS [48]. These pathologies are characterized by the progressive loss of neurons in different areas of the CNS, leading to cognitive, sensory and motor dysfunctions [49]. Other pathological features of neurodegenerative diseases are the accumulation of aggregated proteins, depletion of endogenous antioxidant enzyme activity, mitochondrial dysfunction and increasing neuroinflammation [50]. The link between aging and neuroinflammation has largely emerged and implicates the aberrant modulation of multiple sets of genes and proteins, such as an increased activation of microglia and astrocytes by both NF-kB and cyclooxygenase-2 (COX2) and inducible nitric oxide synthase (iNOS) levels; this, in turn, induces the release of pro-inflammatory cytokines, leading to neuronal death and subsequent cognitive deficits [51].

Considering these aspects, the identification of new molecules and drugs able to prevent, delay or alleviate cognitive impairment characterizing these pathologies appears of great clinical relevance.

Several preclinical studies have suggested beneficial roles for curcumin as an adjuvant therapy in free radical-based diseases [52,53]. As demonstrated in mice, curcumin is metabolized to form several metabolites with relevant biological effects. For its anti-inflammatory, antioxidant, antiproliferative and antimicrobial properties, curcumin has been proposed as an adjuvant treatment of several disorders, including spinal cord injury as well as for neurodegenerative diseases [54,55].

Of particular interest are the potential effects of curcumin in the regulation of nervous system activity. Curcumin can impair extrapyramidal symptoms and increased HO-1 expression through Akt/Nrf2 phosphorylation in the substantia nigra pars compacta of rats treated with rotenone, a pharmacological tool able to destroy dopaminergic neurons and therefore, used to induce experimental Parkinson’s disease (PD) [56]. Curcumin displayed a neuroprotective action by reducing protein misfolding and aggregation through upregulation of heat shock proteins such as Hsp90, Hsp70, Hsp60 and Hsp40 in mice models [57].

Considering its ability to interfere with the apoptotic pathways, curcumin was also demonstrated to exert neuroprotective actions in rats that underwent ischemia/reperfusion injury [58]. In addition, curcumin increased antioxidant molecules GSH and enzymes such as CAT and SOD. It was found that curcumin acts also as ROS scavenging, disrupting amyloid plaques and exhibiting anti-inflammatory and anti-apoptotic effects [59]. The impact of curcumin on neural stems cells and other neural cells is also relevant. In fact, several studies reported the capacity of curcumin to protect neural stem cells [60].

Despite these claimed properties, the poor intestinal absorption, structural instability, limited blood brain barrier (BBB) penetration and rapid degradation of curcumin, limits the potential as a therapeutic agent in clinical trials [61].

The following Table 1, Table 2 and Table 3 summarize the in vitro and in vivo studies of free-curcumin on neurological disorders.

### 3.2. Brain Tumors

Gliomas are the most common primary brain tumors in humans, resulting from glial precursors or astrocytes transformation. GBM is the most frequent, aggressive (grade IV) form of glioma, frequently occurring in the brain or in the spinal cord.

The cellular and molecular origin of GBM is not yet completely known; however, the dysregulation of cellular signaling pathways and several genetic mutations were described as being involved in the regulation of cancer progression, invasion and metastasis formation. The overexpression of epidermal growth factor receptor (EGFR), the hyper-activation of PI3 kinase pathway, mutation of p53 genes or PTEN were reported in GBM formation [89,90].

Despite the progressions in neurosurgery, pharmacological therapy, immune therapy and radiation, the patient’s survival is still poor [91,92].

Interestingly natural compounds and their metabolites received much attention as promising therapeutic agents for the treatment of several human malignancies. Considering the abundant amounts of lipids in brain, the lipophilic nature of curcumin presents a good absorption, availability and stability in the CNS [11].

First, curcumin can inhibit proliferation and induce apoptosis of GBM cell line. The mechanisms require the p53 and caspase 3 activation or decreasing of anti-apoptotic genes, including NF-kB and Bcl2 [93,94]. The decreased expression of bcl-2, and DNA repair enzymes, such as O-6-Methylguanine-DNA Methyltransferase (MGMT), lead to reduced resistance of glioma cells against radiation and chemotherapeutic agents; this suggests that curcumin may be a potential useful adjuvant for common chemotherapeutic agents and radiation [95,96]. It has also been reported that curcumin inhibits migration and invasion in GBM cell line U87MG by modulation of matrix metalloproteinases (MMPs) and decreasing the expression of fascin, a protein involved in F actin aggregation [97].

The GBM cell survival curcumin-induced results decreased in relation to increased ROS production, caspases activation and mitochondrial membrane permeability alteration [98].

Curcumin can also induce autophagy by suppressing the Akt/mammalian target of rapamycin and activates the kinase pathways regulated by extracellular signals [11]. Interestingly curcumin inhibits the viability and proliferation of glioblastoma stem-like cells, considered the subpopulation responsible of GBM recurrence and formation [99,100,101].

In Table 4, the numerous studies on the role of curcumin as potential anti-tumor agent against GBM, are reported.

## 4. Nanotechnological-Based Strategies for Curcumin Delivery and Blood-Brain Barrier Crossing

### 4.1. Preparation and Formulation of Nanocurcumin

The development of nanotechnology-based applications in the health sector offers innovative therapeutic and diagnostic opportunities to address medical needs. In parallel, a need for a specific regulatory framework is growing; no regulatory practice exists for nanotechnology-enabled health products. However, due to the increased complexity of such products and their size-related properties, due to the fast progress in the field and the lack of robust datasets, the question remains whether the identified safety and efficacy requirements of the products are sufficient for a reliable characterization, assessment and market. Thus, new state-of-the-art methods, instruments, approaches or tools must be developed to sufficiently prove their reliability and relevance for the given purpose. Although the preparation methods are not the subject of this work, a brief summary of the main new methods of nano curcumin preparation along with advantages and disadvantages are reported in Table 5. The nanotechnology methods, reviewed by Rai et al., [117], developed to enhance the activity of nanocurcumin are various: coacervation, nanoprecipitation, spray drying, single emulsion, solvent evaporation, microemulsion, wet milling, thin film hydration, solid dispersion, emulsion polymerization, ionic gelation, ultrasonication, antisolvent precipitation and Fessi methods.

Depending on the method of preparation, nanoparticles, nanospheres or nanocapsules can be obtained. Nanocapsules are systems in which the drug is confined to a cavity surrounded by a unique polymer membrane, while nanospheres are matrix systems in which the drug is physically and uniformly dispersed [118,119]. The field of polymer nanoparticles is quickly expanding and playing an important role in a wide spectrum of areas [120]. The polymeric nanoparticles (size between 10–1000 nm) are prepared from biocompatible and biodegradable polymers where the drug is dissolved, entrapped, encapsulated or attached to a nanoparticle matrix.

Curcumin’s low solubility and bioavailability hinder the great properties of this compound and compromise its use in the biomedical field. This limit could be overcome by designing new methods of administration to stabilize the molecule and increase its bioavailability, by reducing its metabolism and increasing the retention time in the bloodstream. In fact, encapsulation of curcumin into nano-carriers (i.e., liposomes, engineered nanoparticles or exosomes, EXOs) and used in preclinical studies for different pathologies, demonstrated an improved efficacy in comparison to free molecule [121,122]. Nano-encapsulation of curcumin improves its stability, avoids the enzymatic and pH degradation, leading to increased half-life [123].

Many different nanotechnology-based strategies (e.g., polymers, liposomes, hydrogels, adjuvants and nanoparticles) have been designed in the last few decades with the aim of efficient curcumin loading, prolonging the time of properties retention, and of inactivation or hydrolysis protection [124]. These nano encapsulated curcumin with improved properties have been tested in vitro, in in vivo experiments and in pre-clinical trials as reported in Table 6.

An interesting strategy is represented by the amorphous solid dispersions (ASD), a modification of the solid state that improves the bioavailability by increasing the rate of dissolution. This technique is based on the incorporation of water-insoluble compounds into a hydrophilic carrier matrix. Zhang et al. [148] demonstrated that ASD forms of curcumin increased cytotoxicity and membrane permeability of U87 glioblastoma cells. In addition, in in vivo studies, the bioavailability of this formulation was 19-fold more efficient compared to free curcumin.

The oral bioavailability of hydroxypropyl methylcellulose-based solid dispersion of curcumin (DW-CUR 20) evaluated in in vivo, exceeded the limits of curcumin in pure form. In a mouse model of acute liver injury induced by tert-butyl hydroperoxide, oral administration of DW-CUR 20 was found to be highly hepatoprotective, as demonstrated by the improvement of the histological liver damage [149].

When curcumin is in form of nanosuspension, in other words, a nanosystem containing only pure drug crystals with a surfactant agent as stabilizer, the saturation and dissolution solubility is higher as the surface area increases. There are two approaches for nanosuspension preparation: top-down and bottom-up [150,151]. In the top-down preparation, particles with a micrometric size are reduced to nano size, while in the bottom-up method the nanosuspension, with a defined size, is formed by precipitation, crystallization, spray drying, et cetera of solutions [152]. The few reports on curcumin nanosuspensions are mainly focused on improved oral delivery. Different surfactants have been used as stabilizers that played a crucial role in the preparation of curcumin nanosuspensions. Curcumin solid lipid nanoparticles (Cur-SLNs) formulate with P-gp modulator excipients, TPGS (D-α-Tocopherol polyethylene glycol 1000 succinate) and Brij78 (that is a docosaethylene glycol mono octadecyl ether), enhance the solubility and bioavailability of curcumin. Again, curcumin nanosuspension prepared by precipitation method with sodium lauryl sulphate and polyvinylpyrrolidone k-60 showed an increased oral solubility, stability and dissolution rate with respect to pure curcumin [153]. High speed homogenization for the nanosuspension formulation by using Arabic gum as a natural polymeric surfactant was reported [154].

Although some polymeric systems are highly biocompatible, adverse long-term administration effects, due to the interaction between nanomaterials and biological systems, could be possible. A valid alternative is the use of lipid-based nanoparticles. They are usually glyceride derivatives that are easily metabolized and their advantage derives from high biocompatibility and biodegradation of lipids.

Liposomes are spherical vesicular systems composed by one or more layers of phospholipids and have an internal aqueous space where the drug can be loaded [155]. De Leo et al. [156] developed a liposome-based formulation for the delivery of curcumin through the colon, in other words, liposome particles with a polymer coating sensitive to pH changes of the gastrointestinal tract. In this study, curcumin was encapsulated in small unilamellar vesicles by micelle-to-vesicle transition method in a simple and organic solvent-free way. Curcumin-loaded liposomes can also be synthesized to provide insights into the influence of thermodynamic characteristics and permeation/dermal penetration of vesicles [157].

Indeed, many other approaches have been investigated as strategies to enhance the therapeutic potential of curcumin, like nanoemulsion, polymeric nanoparticles, microemulsion and miscellaneous nanosystems [158,159,160,161]. The ever increasing need to produce biocompatible nanomaterials and in compliance with the green circular economy, an adequate number of works have developed different bio-based curcumin nanocarriers with specific characteristics and applications, that are summarized in Table 7, modified from [162].

### 4.2. Efficacy of Nano-Encapsulation of Curcumin in Crossing BBB

The poor penetration of curcumin through the BBB together with its chemical-physical characteristics, represent important limitations for the application of these new systems in brain pathologies [163]. The BBB between the blood and the CNS has the physiological function of regulating ionic homeostasis, molecules exchange and cell infiltration, thus preserving the brain microenvironment and protecting the CNS from pathogens or circulating toxins [164,165]. Under pathological conditions, when primary tumors or metastasis grow beyond 1–2 mm in diameter, the BBB is structurally and functionally compromised and becomes blood brain tumor barrier (BBTB) [166]. The exchange of molecules through the BBB is limited by the presence of tight junctions between endothelial cells (ECs), the lack of fenestrations and the low rate of pinocytosis which, in turn, limit the passage through the transcellular way [167,168]. In addition, the expression on ECs of various transporters and multidrug resistance-related proteins (MRPs) can further restrict the entry of many molecules [169].

The structure, function and permeability of BBB can be altered not only by brain cancer, as the GBM, but also in other pathological conditions like MS, epilepsy, acquired immunodeficiency syndrome (AIDS), dementia and stroke [166,167,168,169,170,171]. Although numerous strategies have been designed for crossing the BBB, there are still several concerns that make brain drug delivery difficult. The use of engineered nanocarriers that can protect the drug and allow its transport through the BBB seems to be a winning strategy [172,173]. Nanotechnology allows synthesizing nanodevices with several advantages: (a) selective drug targeting to the diseased tissues; (b) accumulation of higher concentrations of drugs; (c) increase of vascular permeability; (d) BBB crossing; (e) enhancement of drug efficacy and reduction of drug toxicity [174,175,176,177].

For a correct drug-delivery crossing the BBB by using nanofabricated systems, several factors have to be considered such as size, charge, biocompatibility, stability in the blood circulation and controlled release capacity. For example, it has been established that nanoparticles (NPs) with a size smaller than 200 nm and with a positive surface charge cross the BBB more easily [178]. Studies on animal models of CNS diseases favor the use of NPs with a diameter between 50 and 100 nm. No less important is the specificity of nanosystems for BBB, generally determined by functionalizing them with specific proteins. For example, the use of cell penetrating peptides (CPP), penetratin and Tat protein for functionalizing NPs appear to be promising for this purpose. To increase BBB crossing, nanoparticles’ surfaces can be decorated with different ligands for diverse targets such as GLUT1 or albumin transporters, Lf receptors, LRP1 (targeted by angiopep-2) or Tf receptors [179]. In addition, the use of lipophilic and small-sized nanosystems appears to be advantageous, consider their ability to cross the BBB by simple passive diffusion [180]. Last but not least is the morphology of NPs (spherical, cubic, rod-shaped, etc.), which can influence their cellular uptake [181].

The final pathway of nanoparticle entry into the endothelial cells by transcytosis is determined by the initial step of endocytosis. The negative charge of the outer membrane of the endothelial cells affects the entry of the NPs; in fact, positively charged NPs use the adsorbing transcytosis pathway more easily than neutral or negatively charged ones that show a reduced protein adsorption, leading to longer circulation times. This issue can be circumvented by coating the nanoparticles with compounds such as Polyethylene Glycol (PEG) that generates a steric barrier around the nanoparticles avoiding opsonization and their subsequent elimination by the mononuclear phagocyte system [178].

A short review on some of the therapeutic properties of nanomaterials in drug delivery to CNS with patents is reported in Saeedi et al., [180].

Nanoparticles (NPs) can cross the BBB through receptor-mediated transcytosis (RMT), adsorptive-mediated transcytosis (AMT) and by enhanced permeability and retention (EPR) due to compromised tight junctions [173,182,183]. The NPs uptake via RMT needs (i) receptors on ECs of the adluminal (blood) side, (ii) endocytic vesicles moving through cytoplasm of ECs and (iii) the exocytosis of NPs at the abluminal (brain) side. Many receptors can mediate BBB crossing of NPs: transferrin receptor (TR), lactoferrin receptor (LR), insulin receptor (IR), low-density lipoprotein receptor related proteins (LRP), diphtheria toxin receptor, etc. [184,185,186,187,188]. This multivalent system for the treatment of neurodegenerative diseases leads to high local concentration of NPs on brain capillaries and accelerates the BBTB crossing. In AMT, cationic NPs are endocytosed by ECs that present a positive electric charge at their luminal surface and then exocytosed at the abluminal side. The efficacy of the transcytosis transfer via AMT is much higher than RMT, thanks to a greater binding capacity of AMT and to the possible conjugation of NPs with cationic proteins, polyamines or cell penetrating peptides that reinforce the interactions of NPs and ECs [183,189,190,191,192].

**Figure 2:**
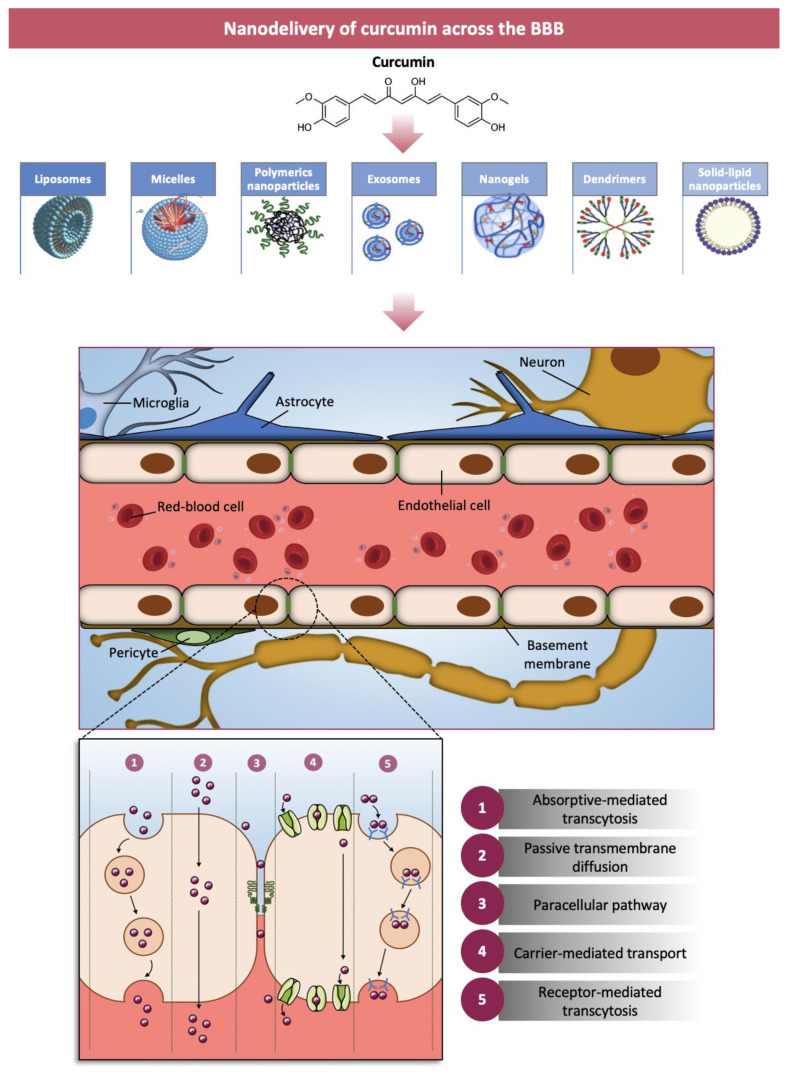
Main types of curcumin nanocarriers, structure of blood brain barrier (BBB) and BBB crossing modality are reported. BBB is composed of endothelial cells (ECs), pericytes (PCs) and astrocytes, whose end-feet cover the basal lamina of the brain capillaries [166,193]. There are several transport routes divided in diffusion and transcytosis by which small soluble molecules and endogenous macromolecules move across the BBB, respectively. Hydrophilic molecules and lipophilic substances diffuse between the cells through the tight junctions or move through the cells by dissolving in the plasma membrane [173]. In addition, carrier mediated transports in which, for example, SLC2/Glut1 glucose and LAT1 amino acid carriers are present, which mediate the uptake of essential nutrients [169]. Transcytosis involves endocytic vesicles shuttling from the luminal cell side to the abluminal side where the molecules are released by exocytosis. The transcytosis process can be specific or receptor-mediated (RMT) [194] and nonspecific or adsorptive-mediated (AMT) [183]. An example of regulated receptor mediated transport is represented by the entry into the ECs of transferrin, insulin and lipoprotein [195].

In Figure 2, the main types of nanostructured curcumin, the BBB structure and the crossing pathways are reported.

### 4.3. Efficacy of Nanoencapsulated Curcumin in the Treatment of Neurodegenerative Diseases

Despite the few in vitro, in vivo experiments and clinical studies, evidences of the bioactive role of different nanoencapsulated curcumin in the prevention and treatment of various CNS-related diseases suggest nanocurcumin as a novel strategy for neurodegenerative AD and PD. Meng et al. [195] investigated the efficacy of a new nanostructured low-density lipoprotein transporter, modified with curcumin loaded lactoferrin for a targeted brain release regulating AD progression. This 100 nm sized nanocurcumin formulation was able to cross the BBB and effectively release curcumin. To examine the effect of nanocurcumin on neuronal loss, 42 amino acid forms of amyloid β peptide (Aβ1-42) were injected bilaterally into the dorsal hippocampi of rats. Histopathological analysis demonstrated the ameliorative effect of the nanostructure on neuronal damage. In addition, plasma levels of malondialdehyde (MDA), an important indicator of lipid peroxidation, decreased in the nanocurcumin treated groups, confirming the BBB crossing and effectiveness in reducing the oxidative damages [73].

In another study, a novel brain-targeting nanoparticle, poly(lactide-co-glycolide)-block-poly(ethylene glycol) (PLGA-PEG) conjugated with B6 peptide and loaded with curcumin (150 nm PLGA-PEG-B6/Cur), was used in in vitro and in vivo tests and demonstrated that nanoparticles could narrow the diameter of curcumin increasing its cellular uptake and blood compatibility [196]. Furthermore, an ex vivo analysis also demonstrated the reduction of hippocampal β-amyloid formation and deposits with a hyper-phosphorylation of tau proteins [197].

The epigenetic regulatory role of patented 120 nm liposomal curcumin formulation, Lipocurc, is expressed by inhibition of DNA methyltransferases (DNMTs), regulation of histone modifications and of miRNAs, binding to DNA and interacting with transcriptional factors in the DJ-1 KO rat model of PD [198]. The DJ-1 knock-out (DJ-1 KO) transgenic rat model of PD was used to test the action of the subchronic treatment with Lipocurc of the transgenic PD rat model, improved motor impairment and reduced apoptosis. Lactoferrin-curcumin nanoparticles played a protective role against rotenone-induced toxicity in SK-N-SH neuroblastoma cells more efficiently than curcumin alone [199].

It is well established that oxidative stress plays a pivotal role in the pathology of PD and it is associated with nitric oxide production and mitochondrial dysfunctions [194]. Encapsulated curcumin in 100 nm sized alginate nanoparticles have been demonstrated to reduce oxidative stress and apoptosis in a transgenic *Drosophila* model [200]. The use of 11.3 nm alginate-curcumin nanoparticles as diet supplement for 24 d in a PD *Drosophila* model caused a strong reduction in oxidative stress-related lipid peroxidation [201].

### 4.4. Efficacy of Nanoencapsulated Curcumin in Glioblastoma Treatment

Numerous studies have reported on the use of curcumin as adjuvant to the GBM treatments [202]. In different experimental models of GBM (HSR-GBM11, JHH-GBM14, hU251MG, U87MG, GL261, F98, C6, N2a GBM cells) treated with different nano encapsulations of curcumin (50 nm polymeric curcumin NanoCur; methoxy polyethylene glycole-poly caprolactone, mPEG-PCL; solid lipid particles, SLCP; curcumin-loaded lipid-core nanocapsules, C-LNCs) obtained common results like the efficient entry by endocytosis, the increase of apoptosis, autophagy, arrest of a G2/M cell cycle, cell proliferation or GBM neurospheres formation and anti-tumorigenesis effects, which were better than for free curcumin [93,94,203,204,205]. An interesting approach, coupling antibody-conjugated biodegradable Poly (D, l-lactic-co-glycolic acid) nanoparticles (250 nm PLGA NPs) to enhance the photodynamic efficiency of curcumin on DKMG/EGFRvIII cells (EGFRvIII overexpressed human glioblastoma cell line), is very efficient at inducing curcumin release, cell internalization, cytotoxicity and photo-toxicity in GBM cells [206].

Furthermore, 80 mg of curcumin encapsulated into nano micelles is able to suppress U373 cell growth by modulation of Wnt and NF-κB pathways, and determined early G2/M cell cycle arrest followed by an increased sub-G1 and cell death induction [207]. Another novel nano encapsulation of curcumin, called dendrosomal curcumin (DNC), inhibits U87MG cell proliferation in a time- and dose-dependent manner that, when synergistically administered with p53 overexpression, enhances the number of apoptotic cells. Similar accumulation of apoptotic U87MG in SubG1 phase in a time- and dose-dependent manner was also observed with spherical 142 nm size DNC, by downregulation of octamer binding protein 4 (OCT4) and SRY (sex determining region Y)-box 2 (SOX-2) transcription factors [208]. The simultaneous p53 overexpression and DNC administration enhanced growth arrest and DNA damage (GADD45) gene expression and reduced NF-κB and c-Myc expression [209].

In a study of thirteen glioblastoma patients orally administered with 70 mg of 142 nm micellar curcuminoids (57.4 mg curcumin, 11.2 mg demethoxycurcumin (DMC), and 1.4 mg bis-demethoxycurcumin (BDMC)) three times per day for 4 days (total amount of 689 mg curcumin, 134 mg DMC and 17 mg BDMC) before surgery, led to quantifiable curcuminoids inside glioblastomas and may alter intratumoral energy metabolism [210].

Compared to free curcumin, in vivo treatment of mice with 196 nm C-LNCs results in a greater decrease in brain tumor size and prolonged survival [211]. Antibody-linked curcumin or phytosomal curcumin (curcuminoids (CC)) formulation prepared by binding a defined mass of CC to an equimolar mass of phosphatidylcholine that wrap and protect the CC hydrophobic domains around allowing in vivo higher bioavailability and stability) treatment causes complete remission in about 60% of GBM mice models, probably by inducing the shift of macrophages from the tumor-promoting M2 type to tumoricidal M1 type, the activation of NK cells and apoptotic cell death [212,213,214].

Finally, nano encapsulation of curcumin in polysaccharide matrices (diameters range 210–240 nm) based on hyaluronic acid (HA), chitosan hydrochloride (CSH) and curcumin-lactoferrin conjugated (Lf-Cur-PSNPs) show good BBB penetration. Lf-Cur-PSNPs are preferentially taken up by brain capillary endothelial cells and, after crossing the BBB, remain intact and more effective in targeting C6 glioma cells. Polyelectrolyte complex nanoparticles (PENPs) based on hyaluronic acid/chitosan (HA/CS) as carriers for water-insoluble curcumin as therapeutic carrier for brain gliomas, showed strong dose dependent cytotoxicity and high performance uptake by C6 glioma cells, through active endocytosis, macropinocytosis and clathrin-, caveolae- and CD44-mediated endocytosis [215].

### 4.5. Exosomes as Novel Delivery System for Curcumin

In recent years, novel nano-sized natural vesicles, the exosomes (EXOs), are receiving a lot of interest for their cell targeting potential, biocompatibility, long circulation time, capability to cross biological barriers and to penetrate into deep tissue [216,217,218].

EXOs (30–150 nm size) are the smallest members of the extracellular vesicle (EV) family [219]. EXOs are formed by inward budding of the endosomal membranes of intraluminal vesicles (ILVs) to form multivesicular bodies (MVBs). After maturation, MVBs fuse with the plasma membrane and release ILVs in the form of EXOs into the extracellular environment, where they can mediate intercellular communication, exchange materials with other cells, eliminate unwanted cell products and mediate immune surveillance [220]. EXOs are composed of proteins, lipids and nucleic acids (DNA, mRNA, miRNA, lncRNA, etc.) stored during formation. EXOs are present in body fluids, such as blood, cerebrospinal fluid, amniotic fluid, urine, milk, etc., and participate in various physiological and pathological functions [221]. Recently, the presence of EXO-like vesicles, whose biogenesis is still unknown, have also been described in plants [222]. EXOs can be isolated, purified and characterized, following procedures that are continuously developing in parallel with the technologies for their loading with bioactive compounds [223,224].

**Figure 3:**
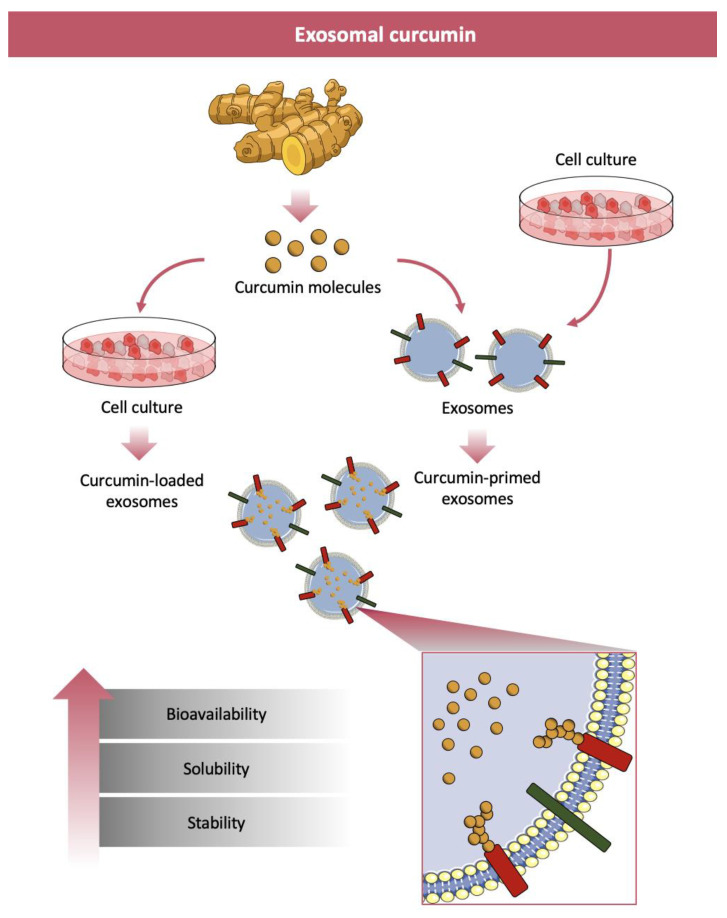
Synthesis of exosomes-loaded and exosomes-primed curcumin and benefits in terms of bioavailability, solubility and stability achieved in curcumin delivery.

Therapeutic drugs are loaded into EXOs by an active or passive method, with different loading yields and stabilities. Sonication, extrusion, electroporation or freeze-thaw cycles methods that disrupt EXOs membrane, restored after the loading process, are the main methods for active cargo loading [225]. Active loading (or EXOs loading) increases cargo up to 11 times compared to passive loading [226], but for the intrinsic characteristics of the process the native EXOs structure and targeting features are damaged. Passive cargo loading (or EXOs primed) consists of incubation of bioactive compounds with isolated EXOs or incubation of bioactive compounds with donor cells before vesicle isolation [225]. The first passive encapsulation process is based on the molecule diffusion through EXOs lipid membrane and strictly depends on the hydrophobic or hydrophilic properties of molecules to be loaded. The second passive encapsulation process is less common and consists, first, in the incubation of cargo with donor cells followed by isolation of EXOs engineered with the specific cargo at the cellular level under natural conditions [225]. In Figure 3 the synthesis of exosomes-loaded and exosomes-primed curcumin and the benefits in terms of bioavailability, solubility and stability achieved in curcumin delivery are reported.

Two recently published reviews discuss the use and applications of plant and animal derived EXOs that could potentially solve most of the problems of the existing nano-delivery systems [222,223].

EXOs can bind hydrophobic molecules that, as a consequence, increase their transport and their bioavailability allowing an efficient cellular uptake. Curcumin, that is a hydrophobic molecule, can be bound and transported by EXOs. This incorporation increases curcumin solubility, stability and bioavailability when compared to others and thus encourages the use of EXOs for therapeutic purposes [227,228,229,230,231,232,233].

Both EXOs curcumin loaded or primed have in vitro and in vivo therapeutic effects: anti-hypocholesterolemia, anticancer and anti-inflammatory (in cancer, ischemia-reperfusion injury and ischemic stroke). Table 8 reports a summary of anticancer effects of exosomes encapsulated curcumin.

Despite the mechanisms of how EXOs reaches the brain still being unknown, they are considered promising therapeutic agents for brain protection. Indeed, intranasal administration of EXOs-encapsulated curcumin in LPS-induced brain inflammation and in autoimmune encephalomyelitis in C57BL/6j induced a reduction in the number of activated inflammatory microglial cells [234]. The treatment with EXOs-encapsulated curcumin of stroke injury in type-1 diabetic mice ameliorates neurodegeneration, promotes neuronal survival and restores cognitive function by reducing infarct volume, edema and vascular damage [235]. In a model of mouse cerebral ischemia, treatment with EXOs-encapsulated curcumin reduced pro-inflammatory cytokines TNF-α, IL-1β and IL-6 levels and efficiently suppressed inflammation and cellular apoptosis in the lesion region of the ischemic brain [236]. Curcumin primed EXOs, prepared by incubating mouse brain endothelial cells with 7.5 µM curcumin for 72 h protected brain endothelial cell layer permeability by decreasing oxidative stress and mitigating impaired junction proteins [237]. Curcumin-loaded exosomes, isolated from mouse embryonic stem cells and administered nasally to ischemia-injured mice, was distributed into all brain regions and improved the neurological score after three days of treatment. In addition, the treatment restored vascular endothelial tight (claudin-5 and occludin) and adherent (VE-cadherin) junction proteins, suggesting that combining the potentials of embryonic stem cell exosomes and curcumin can help neurovascular restoration following ischemia-reperfusion injury in mice [238].

**Table 8:** In vitro studies on exosomes encapsulated curcumin effect in cancer.

Disease	Exosomes Origin	Exosomes Target	Outcomes	Ref.
Pancreatic cancer	PANC-1 cells, MIA PaCa-2 cells	PANC-1 cells MIA PaCa-2 cells	Cell death induction	[239]
Lung, breast, and cervical cancers	Raw bovine milk	H1299, A549 lung cancer cells MDA-MB-231, T47D breast cancer cells, HeLa cervical cancer cells	Antiproliferative, anti-inflammatory, and antitumor activities	[227]
Breast cancer	TS/A, 4T.1, B16 tumor cells	Murine mammary adenocarcinoma of spontaneous BALB/c origin	Anticancer properties by inhibition of NK cell activity	[240]
Lung cancer	H1299	TCF21, BEAS-2B, A549, PC9, H1299Mouse lung cancer cells	Anti-cancer function by downregulating DNMT1, thereby upregulating TCF21	[241]
Chronic myelogenous leukemia	K562, LAMA84	CML mouse model	Cur-Exo containing miR-21 indicated an antineoplastic effect in chronic myeloid leukemia	[242]
Chronic myelogenous leukemia	K562, LAMA84	CML mouse model	Angiogenic effect	[243,244]
Lymphoma Mammary gland cancer, Colon cancer	Grapefruit	HUVEC/human and T-lymphoma EL4 cells, 4T1 and 4TO7 breast cancer cell lines, NMuMG mammary gland epithelial cells, CT26 colon cancer/mouse	Inhibition of breast tumor and colon tumor growth	[245]

## 5. Conclusions

Nanotechnology is fundamental in developing new strategies aimed to improve bioavailability, stability and targeting of nutraceuticals, for those, like curcuminoids, that possess low absorption and are rapidly metabolized and eliminated. Among the nutraceuticals, the pleiotropic biological activities of curcumin hold great promise for application in the clinics and for the global nutraceutical products market, that is estimated will grow about 7.2% in the next five years. The antioxidant, anti-inflammatory and anticancer activities of curcumin provide diverse health benefits against a wide spectrum of human diseases. Therefore, companies involved world wide in the nutraceuticals markets are developing new technological strategies to improve the beneficial effects for human health. In this review, we have discussed the advantageous use of curcumin against the central nervous system related disorders as well as the problems connected to its poor aqueous solubility and low bioavailability and to the fact that under various conditions curcumin is unstable, easily degraded or metabolized. For this purpose, in the field of neurological disorders (neurodegenerative diseases and cancer), we have further discussed the scientifically verified benefits of curcumin impaired by low brain bioavailability due to the limited blood-brain barrier permeability. Then, different kinds of curcumin nano-carriers (such as nanoparticles, micelles, dendrimers, conjugation with other materials, bio-based nanomaterials) are discussed along with their characteristic features like enhanced stability, bioavailability, solubility, in vivo uptake, bioactivity and safety. Among the recent research directions, a special focus has been directed at EXOs for their excellent properties, in other words, high loading efficiency and exact offloading process. Overall, the nanocurcumin delivery may lead to a significant improvement of its efficacy in CNS related disorders. However, in future, further in vivo and clinical investigations will help to achieve a safe and beneficial administration to AD, PD, ALS and cancer patients.

## Figures and Tables

**Table 1:** In vitro and in vivo studies of curcumin on Parkinson’s disease.

Cell Line /Animal Model	Route of Treatment Dose Duration	Outcomes	Ref.
ICR mice	Intravenous injection 200 mg/kg 7 days	Increase in SOD1 expression; inhibition of glial response; reduction in the activation of astrocytes	[62]
C57BL/6J mice derived primary mesencephalic astrocyte	Diet 40 mg/kg 7 weeks	Reduction of ROS; inhibition of CYP2E1 activity; enhancement of GDNF and TGFβ1 expression	[63]
C57BL mice	Diet 0.5% or 2.0% (*w*/*w*) 7 weeks	Block of the neurotoxicity of MPTP in the nigrostriatal dopaminergic system; increased expression of GDNF and TGFβ1	[64]
SH-SY5Y neuronal cells	100, 200, and 300 μM curcumin for 20 h	Modulation of α-synuclein aggregation and toxicity	[65]
SH-SY5Y neuronal cells	30 μM 1 day	Neuroprotection by inducing macroautophagy	[66]
C57BL/6 mice	Intraperitoneal injection 24 mg/kg 7 days	Neuroprotection by enhancement of monoamine transporter expressions and cosseted mitochondria	[67]
Sprague-Dawley (SD) rats	Gavage 10 and 15 μmol/L 3 weeks	Activation of the Wnt/β-catenin signaling pathway; increase in glutathione peroxidase and superoxide dismutase	[68]

**Table 2:** In vitro and in vivo studies of curcumin on Alzheimer’s disease.

Cell Line /Animal Model	Route of Treatment Dose Duration	Outcomes	Ref.
APPV717I transgenic mice.	Diet 40 mg/kg 4 weeks	Reduction of amyloid-β protein and inhibition of inflammation	[69]
APPswe/PS1dE9dtg mice	Diet high (400 mg/kg medium (200 mg/kg) low (100 mg/kg) 6 months	Reduction of the expressions of hippocampal Aβ40, Aβ42 and ADDLs	[70]
hTau transgenic mice	Diet 500 ppm 19–20 months	Reduction of soluble Tau oligomers and Fyn, perhaps through increasing HSP70, HSP90, and HSC70. Improvement of the excitatory synaptic profile.	[57]
Tg2576	Diet low (160 ppm) high (5000 ppm) 6 months	Decrease of insoluble β-amyloid (Aβ), soluble Aβ, and plaque. Suppression of microgliosis	[71]
Wistar rats	Gavage 25, 50,100 mg/kg 30 days	Increase of neurogenesis Decrease of neuroinflammation	[72]
Amyloid-beta (Abeta) peptide-infused rats	Diet 3 mg/kg	Spatial memory enhancement	[73]
APPswe/PS1dE9 mice	Intravenous 7.5 mg/kg/day 7 days	Reverse in existing amyloid pathology and associated neurotoxicity; Prevention of oxidative stress, inflammation and neurotoxicity	[74]
SH-SY5Y neuronal cells		Neuroprotection against Aβ-induced mitochondrial metabolic deficiency; abnormal alteration of oxidative stress	[75]
Rats primary adult and fetal neurons	3.3 ± 0.4 and 8 ± 1 μM	Inhibition of oligomerization of tau and disaggregation of tau filaments	[76]
APPswe/PS1Δ9 mice	Intraperitoneal injection 50 mg/kg 4 weeks	Reduction of activation of microglia and astrocytes cytokine production and inhibition of nuclear factor kappa B (NF-κB) signaling pathway	[77]
Tg2576 mice	Diet 4 g/kg 12 weeks	Suppression of neuroinflammation via regulation of cPLA2/LPC signaling pathways and inhibition the cytokine IL-1 and GFAP	[78]
APP transgenic mice (line J20)	Gavage 0.75 mg/mL 90 days	Enhancement of amyloid clearance and modulation of neuroinflammation	[79]

**Table 3:** In vitro and in vivo studies of curcumin on multiple sclerosis.

Cell Line /Animal Model	Route of Treatment Dose Duration	Outcomes	Ref.
SJL/ J mice	Intravenous injection 50 or 100 μg 25 days	Amelioration of severity and duration of clinical paralysis Decrease of inflammation and demyelination in the CNS	[80]
MBP-immune spleen cells	20 μg/mL	Decrease of proliferation of Th1 cell, IFN-∂ and IL-12 production	[80]
C57BL/6 and BALB/c generated DC	25 μM	Decrease of expression of CD80, CD86, and MHC class II molecules Inhibition of the maturation of DC, secretion of IL-12 and Th1 activation	[81]
Peripheral blood mononuclear cells (PBMC)	20 μg/mL 18 h	Decrease of IL-12 -induced STAT4 phosphorylation, IFN-∂ production, and IL-12 Rβ1 and β2 expression increase of IFN-β-induced STAT4 phosphorylation, IFN-α-induced IL-10, and IFNAR1 expression	[82]
C57BL/6 and SJL/ J mice	Diet 100 μg 14 days	Amelioration of symptoms and intensity Inhibition of neural antigen-specific T cell response	[83]
C6 rat astrocytoma cells	2.5, 10, 25 μM 30 min	Decrease of expression of CCL2 mRNA and protein Downregulation of CCL2 expression	[84]
Albino Wistar rats	Oral 60 mg/kg 3 weeks	Protection against neuronal degeneration	[85]
Primary microglia cultures from P3-P6 Sprague-Dawley rats		Protect axons from NO-mediated degeneration	[86]
U373-MG human astrocytes	0, 2.5, 5 μM	Decrease of release of IL-6 and MMP-9 Downregulation of MCP-1 mRNA expression	[87]
Lumbar spinal cord	12.5 mg/kg	Decrease of demyelination, immune cells infiltration, IL-17, NF-κB, and TNF-α Receptor expression increase of expression of IL-4 and foxp3	[88]

**Table 4:** Anti-tumor effects of curcumin on glioblastoma cell lines.

Cell Line	Dose	Outcomes	Ref.
A172	10 µM	Autophagy induction	[102]
U87MG	10 µM	Migration and invasion decrease	[97]
U87MG, U251MG	10 µM	Apoptosis induction	[103]
U87MG, GL261,P98, C6-glioma N2a	25 µM	Autophagy induction	[93,94]
A172	50 µM	Apoptosis induction	[104]
U118MG, U87MG, U251MG	20 µM	Proliferation and migration inhibition Apoptosis induction	[105]
C6	10 µM	Tumor growth inhibition Apoptosis induction	[106]
Glio 3, 4, 9, 11, 14	25 µM	Proliferation inhibition ROS induction	[107]
U87MG, U251MG	10, 20, 40 µM	Proliferation and migration inhibition Apoptosis induction	[96]
GB3B, GB4B, GB8G	46.4 µM	Cell death induction	[108]
U87MG	20, 40 µM	Cell cycle arrest and apoptosis induction Proliferation inhibition	[109]
U87MG	20, 100 µM	Proliferation inhibition Apoptosis induction	[110]
U373MG	50 µM	Proliferation and migration inhibition Cell death induction	[111]
A172, MZ-18, MZ-54, Mz-256, Mz-304	10, 20, 50 µM	Proliferation, migration and invasion inhibition	[112]
DBTRG	10, 20, 30 µg/mL	Cell cycle arrest Apoptosis induction	[113]
U87MG, C6	5, 10, 20 µM/L	Cell cycle arrest Proliferation inhibition Apoptosis induction	[114]
T98G, U87MG, T67, C6	25–50 µM/L	Cell growth and chemotherapy resistance suppression	[95]
U87MG, T98G	25, 50 µM	Apoptosis induction	[115,116]

**Table 5:** Main procedures for obtaining nanocurcumin formulations, their advantages, disadvantages and main features, obtained by summarizing data reported in [118].

Method	Procedure	Advantages	Disadvantages	Size Range (nm)	Encapsulation Efficiency (%)	Release Efficiency (%)
Coacervation technique	Dissolution of polymers in organic solvent. Suspension of curcumin, stirring and mixing. Centrifugation	Inexpensive Absence of hazardous solvents	Require large amounts of solvent	87–600	45	90 after 10 d
Nanoprecipitation	Dissolution of polymers in organic solvent. Suspension of curcumin, stirring and mixing in water.	Facility to develop nanoparticles in one step, not much expense is involved, low electric power is required, and it is fast	The success of preparing nanoparticles is restricted only to a narrow region of the polymer/solvent/anti-solvent composition map	125–500	90	90 after 12 h
Spray-drying method	Curcumin and polymers are dissolved in the same mixture of solvents. Evaporation of solvents by hot air flow.	Rapid, continuous, cost-effective, reproducible, single-step and scalable process	Low yield	125–750	90	90 after 8 h
Solvent evaporation method	Preparation of a solution consisting of polymer and curcumin. Evaporation of solvent used for dissolving curcumin.	Prevention of thermal deposition by using low temperature in evaporation step	Expensive use of reagents. Time-consuming evaporation process	90–120	80	80 after 72 h
Microemulsion	Stirring on surfactant and adding of curcumin along oil and water.	Very high increase of curcumin biological activity. Easy method	Very sensible to temperature and pH variation during synthesis	2–100	80	90 after 8 h
Emulsion polymerization method	Surfactant is dissolved in pure water by ultrasonication. Curcumin is dissolved in organic solvent and added to the surfactant	Fast and readily scalable method	Not reported	85–200	70	90 after 24 h

**Table 6:** Summary of some types of nano encapsulation of curcumin.

Type of Nanocarrier, Form and Size (nm)	Type of Disease	Outcomes	References
Globular liposomes 25–205	Breast, colorectal and lung cancer Melanoma Renal ischemia Malaria	High curcumin solubility, stability and tissue distribution. Increased anti tumor and anti angiogenesis effect. Anti malarial effect	[125,126,127,128,129,130]
Spherical micelles 10–100	Lung, colorectal and breast cancer	High curcumin solubility, bioavailability. Anti oxidative and anti tumor effect.	[131,132,133,134]
Cyclic cyclodextrins 150–500	Bowel disease Breast, lung, pancreas and prostate cancer	High curcumin solubility, bioavailability. Enhanced anti proliferative effect. Anti cancer and anti inflammatory effect.	[135,136,137]
Globular dendrimers 15–150	Breast and colon cancer	High curcumin stability. Enhanced anti proliferative and anti cancer effect.	[138,139]
Cross-linked polymer network nanogel 10–200	Melanoma Breast, pancreas, colorectal and skin cancer	High curcumin bioavailability, half-life and controlled release. Anti cancer effect.	[140,141,142]
Globular gold nanoparticles 200–250	Prostate and colorectal cancer.	High curcumin stability. Enhanced anti proliferative and anti cancer effect.	[143,144]
Spherical solid lipid nanoparticles 50–1000	Cerebral ischemia Colitis Allergy Breast cancer	Prolonged blood circulation Anti inflammatory effect Improved brain delivery	[145,146,147]

**Table 7:** In vivo and in vitro applications of bio-based curcumin nanocarriers.

In Vitro/In Vivo /Simulated System	Properties	Nanocarrier
HCT116 cells	Protection of antioxidant properties Cytotoxicity, cell cycle arrest	Cu loading EWP Cu-PECs
Caco-2 cells	Improved permeability efficiency	Cu-ACRU/CS NPs
	Improved hydrophobic drug delivery	Cu-loaded RBA-CS NPs
	Improved antioxidant activity, stability	Cu-loaded BSA-dextran NPs
	Improved stability and bioaccessibility	CDG-CANPs
HeLa cells	Improved solubility and therapeutic efficacy	Cu-loaded gel-mPEG nanogels
	Improved bioavailability, uptake and controlled release. Anticancer potential	OSA starch loaded nano Cu
HeLa cells H9c2 cells	Enhanced cytotoxicity	Cu-Alg NPs
HaCaT cells	Improved drug release and transdermal penetration	Cu-Chitosan NPs
	Improved uptake	Cu-CS/Alg NPs
L929 cells MCF-7 cells	Killing of cancer cells Inhibition of microbial growth	ANC NPs
HCT116 cells MCF-7 cells	Improved antiproliferative activity	SSPS NPs
MCF-7 cells	Improved therapeutic efficacy	BSA@CUR NPs
Kelly cells	Improved cytotoxicity	Cu-loaded silk fibroin NPs
A549 cells	Improved uptake and cytotoxicity	Cu cross-linked HAS NPs
In vitro model of osteoarthritis	Improved cyto and hemo compatibility	Cu plus SFNs
Rats	Improved plasma circulation time	Cu-loaded silk NPs
	Improved anti-bacterial growth and hair follicles growth	Cu-loaded film
Mice	Reduction of pro-inflammatory cytokines in skin	Cu-NLCs
Murine model of melanoma	Improved survival and reduction of tumor size	Cu-loaded BSA NPs
Simulated GIT	Improved protection of hydrophobic drug	Cu-zein/rhamnolipid complexes
Simulated gastro-intestinal digestion	Improved stability and control release	Zein-HA NPs
	Improved antioxidant activity	Pectin coated CZ NPs
Simulated gastric and intestinal fluids	Improved encapsulation efficiency	Starch NPs
GIT model	To be exploited for functional food and beverage	Cu-loaded zein NPs

Abbreviations: HAS, human serum albumin; BSA, bovine serum albumin; NPs, nanoparticles; HA, hyaluronic acid; Apt-HSA/CCM, aptamer-decorated curcumin-loaded human serum albumin; SSPS, soluble soybean polysaccharide; Cu-ACRU/CS, curcumin-loaded acylated cruciferin/charged chitosan; CDG-CANPs, curcumin diethyl diglutarate-loaded chitosan/alginate NPs; Cu-AlgNP, curcumin loaded alginate NP; Cu-CS/Alg NPs, curcumin-loaded chitosan/alginate NPs; CMC, carboxymethyl cellulose; Cu-NLCs curcumin loaded nanostructured lipid carriers; ANC, aminated nanocellulose; EWP, egg white protein; PECs, polyelectrolyte complexes; SC, sodium caseinate; SA, sodium alginate; GIT, gastro-intestinal tract.
